# Fabrication of low-temperature solid oxide fuel cells with a nanothin protective layer by atomic layer deposition

**DOI:** 10.1186/1556-276X-8-48

**Published:** 2013-01-23

**Authors:** Sanghoon Ji, Ikwhang Chang, Yoon Ho Lee, Joonho Park, Jun Yeol Paek, Min Hwan Lee, Suk Won Cha

**Affiliations:** 1 Graduate School of Convergence Science and Technology, Seoul National University, 864-1 Lui Dong, Yeongtong-Gu, Suwon, Gyeonggi-Do, 433-270, South Korea; 2 School of Mechanical and Aerospace Engineering, Seoul National University, 599 Gwanak-ro, Gwanak-gu, Seoul, 151-742, South Korea; 3 School of Engineering, University of California, Merced, 5200 North Lake Road, Merced, CA, 95343, USA

**Keywords:** Atomic layer deposition, Protective layer, Thin-film solid oxide fuel cell, Yttria-stabilized zirconia, Gadolinium-doped ceria, Anodic aluminum oxide

## Abstract

Anode aluminum oxide-supported thin-film fuel cells having a sub-500-nm-thick bilayered electrolyte comprising a gadolinium-doped ceria (GDC) layer and an yttria-stabilized zirconia (YSZ) layer were fabricated and electrochemically characterized in order to investigate the effect of the YSZ protective layer. The highly dense and thin YSZ layer acted as a blockage against electron and oxygen permeation between the anode and GDC electrolyte. Dense GDC and YSZ thin films were fabricated using radio frequency sputtering and atomic layer deposition techniques, respectively. The resulting bilayered thin-film fuel cell generated a significantly higher open circuit voltage of approximately 1.07 V compared with a thin-film fuel cell with a single-layered GDC electrolyte (approximately 0.3 V).

## Background

Solid oxide fuel cells (SOFCs) normally operate at considerably high temperatures (>700°C) to facilitate ionic charge transport and electrode kinetics [1,2]. Encountered by issues such as limited material selection and poor cell durability, many researchers have tried to reduce the operating temperature [3-5]. However, lower operating temperature led to a significant sacrifice in energy conversion efficiency due to the resulting increase in ohmic and activation losses [1].

There are roughly two ways to minimize the ohmic loss surging at lower operating temperatures. One is to reduce the thickness of the electrolyte, and the other is to synthesize materials with higher ionic conductivities. First, the strategy to reduce in electrolyte thickness has been carried out by many research groups [6-10]. Shim et al. demonstrated that a fuel cell employing a 40-nm-thick yttria-stabilized zirconia (YSZ) can generate a power density of 270 mW/cm^2^ at 350°C [11], while Kerman et al. demonstrated 1,037 mW/cm^2^ at 500°C from a 100-nm-thick YSZ-based fuel cell [12]. Another approach of minimizing ohmic loss is using electrolytes with higher ionic conductivities. Gadolinium-doped ceria (GDC) has been considered as a promising electrolyte material due to its excellent oxygen ion conductivity at low temperatures [13,14]. However, the tendency of GDC being easily reduced at low oxygen partial pressures makes its usage as a fuel-cell electrolyte less attractive because the material will have a higher electronic conductivity as it is reduced. For this reason, many studies have been performed to prevent electronic conduction through GDC film by placing an electron-blocking layer in the series [15-17]. Liu et al. demonstrated the electron-blocking effect of a 3-μm-thick YSZ layer in a thin-film fuel cell with a GDC/YSZ bilayered electrolyte [18]. If the GDC electrolyte thickness was reduced down to a few microns, another problem emerges, i.e., oxygen gas from the cathode side starts to permeate through the thin GDC electrolyte [13,19]. For the reasons mentioned, the application of a protective layer is essential for GDC-based thin-film fuel cells. Recently, Myung et al. demonstrated that a thin-film fuel cell having a 100-nm-thick YSZ layer deposited by pulsed laser deposition onto a 1.4-μm-thick GDC layer actually prevented both the reduction of ceria at low oxygen partial pressures and oxygen permeation across the GDC thin layer [20]. For the development of large-scale thin-film fuel cells, an anodic aluminum oxide (AAO) template has been considered as their substrate due to its high scalability potential. However, commercially available AAO templates have a considerably rough surface unlike silicon-based substrates, which have been used for conventional thin-film fuel cells. For this reason, atomic layer deposition (ALD) technique was employed to deposit a highly conformal and dense YSZ layer to minimize uncontrolled pinholes and/or morphological irregularities.

In this report, we demonstrate a prototypical, AAO-supported thin-film fuel cell with a bilayered electrolyte comprising a GDC film and a thin protective YSZ layer. The radio frequency (RF)-sputtered GDC layer with excellent oxygen ion conductivity is used as the primary electrolyte layer, while the YSZ layer deposited by ALD technique prevents the reduction of ceria at low oxygen partial pressure and oxygen permeation across the GDC thin layer. To investigate the effect of ALD YSZ layer as a protective layer, the electrochemical performance of a GDC/YSZ bilayered thin-film electrolyte fuel cell is compared with that of a single-layered GDC-based thin-film fuel cell.

## Methods

### Thin-film characterization

Chemical composition of thin films was analyzed by X-ray photoelectron spectroscopy (XPS) (AXIS Hsi, Kratos Analytical, Ltd., Manchester, UK). Possible surface contamination was eliminated by 150 eV of Ar-ion etching for 30 s prior to XPS analysis. The microstructure of thin films was investigated using focused ion beam and field emission scanning electron microscopy (FE-SEM) (Quanta 3D FEG, FEI Company, Hillsboro, OR, USA), and a few nanometer-thick Pt layer was coated on samples to prevent thin films from being etched by FE-SEM imaging.

### Electrochemical evaluation

A test cell was attached to a custom-made hydrogen feeding chamber using a ceramic adhesive (CP4010, Aremco Products, Inc., Briarcliff Manor, NY, USA) and heated to 450°C using a halogen heating system. Dry H_2_ gas with a mass flow of 25 sccm was supplied to the anode side, and cathode was exposed to atmospheric environment. Anode was connected to a silver wire, and cathode was contacted by a hardened steel probe. Polarization of thin-film fuel cells was analyzed using an electrochemical testing system (1287/1260, Solartron Analytical, Hampshire, UK).

## Results and discussion

### Thin-film electrolyte fabrication

**Figure 1:**
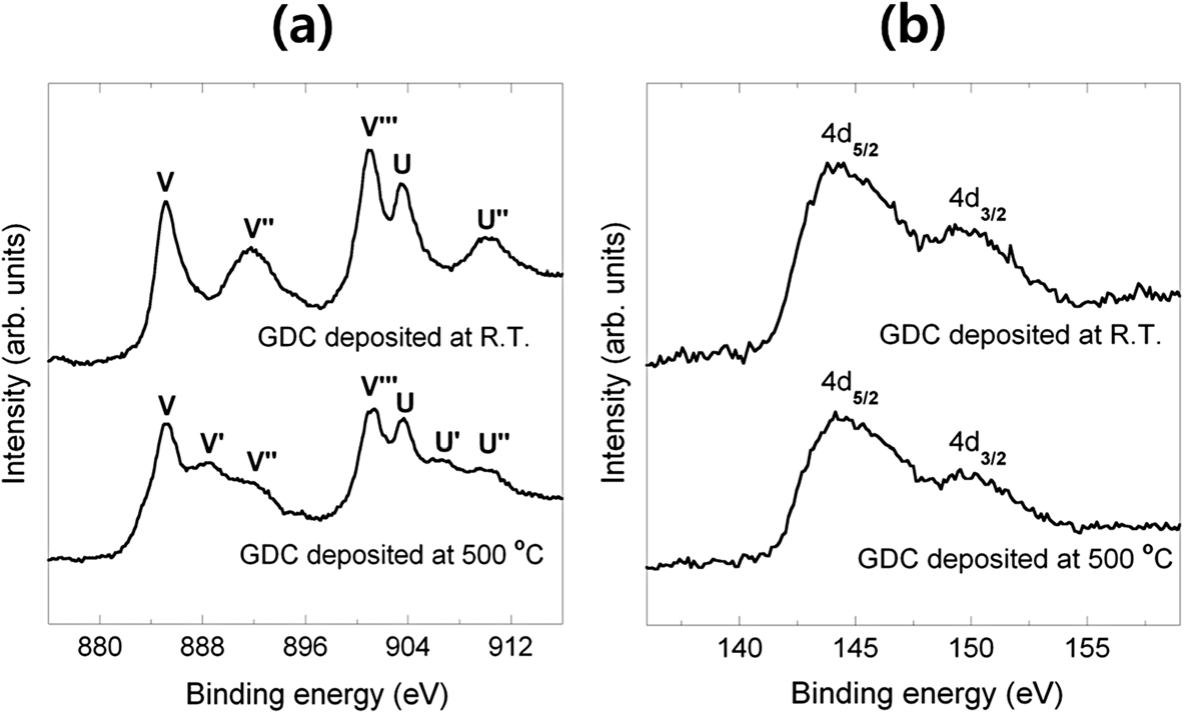
**XPS spectra of (a) Ce 3*d *and (b) Gd 4*d *core levels of GDC thin films.**

GDC thin-film was fabricated by a commercial sputter (A-Tech System Ltd., Incheon, South Korea). Gd-Ce alloy (with 10 at.% Gd) was used as the GDC target. Target-to-substrate (T-S) distance was 80 mm. GDC thin films were deposited at a mixed Ar/O_2_ gas pressure of 5 mTorr. Volume fraction of O_2_ to Ar was 0.2. RF power was set at 150 W. The growth rates of GDC thin films deposited at 100°C and 500°C were approximately 42 and 20 nm/h, respectively. Considering that the packing density of GDC thin-film increases as the substrate temperature increases [21], the substrate was heated to a high temperature of 500°C [1] in order to accommodate more volume for bulk ionic conduction. To determine the chemical composition of GDC thin films, XPS analysis was carried out. A GDC thin-film deposited at 500°C (GDC-H) was compared to a film prepared at room temperature (GDC-R). Figure 1a,b respectively shows the XPS spectra of Ce 3*d* and Gd 4*d* core levels of GDC-R and GDC-H. As shown in Figure 1a, the Ce 3*d* core level of GDC-R did not show spin orbital doublets (*V*^′^, *U*^′^) unlike GDC-H, which is a characteristic of the Ce^3+^ binding state [22]. This result reveals that GDC-H contains reduced cerium oxide (e.g., Ce_2_O_3_) as well as cerium dioxide. The Gd 4*d* core level in Figure 1b illustrated characteristic peaks that are very similar to those of gadolinium oxide, and there was no distinct difference between the two samples. As for atomic concentrations, GDC-H had a higher Gd doping concentration (Gd 4*d* ≈ 13%) than the GDC target (approximately 10%). It is tentatively attributed to the fact that cerium oxide with a lower molecular weight becomes more volatile than gadolinium oxide as substrate temperature increases [23].

**Figure 2:**
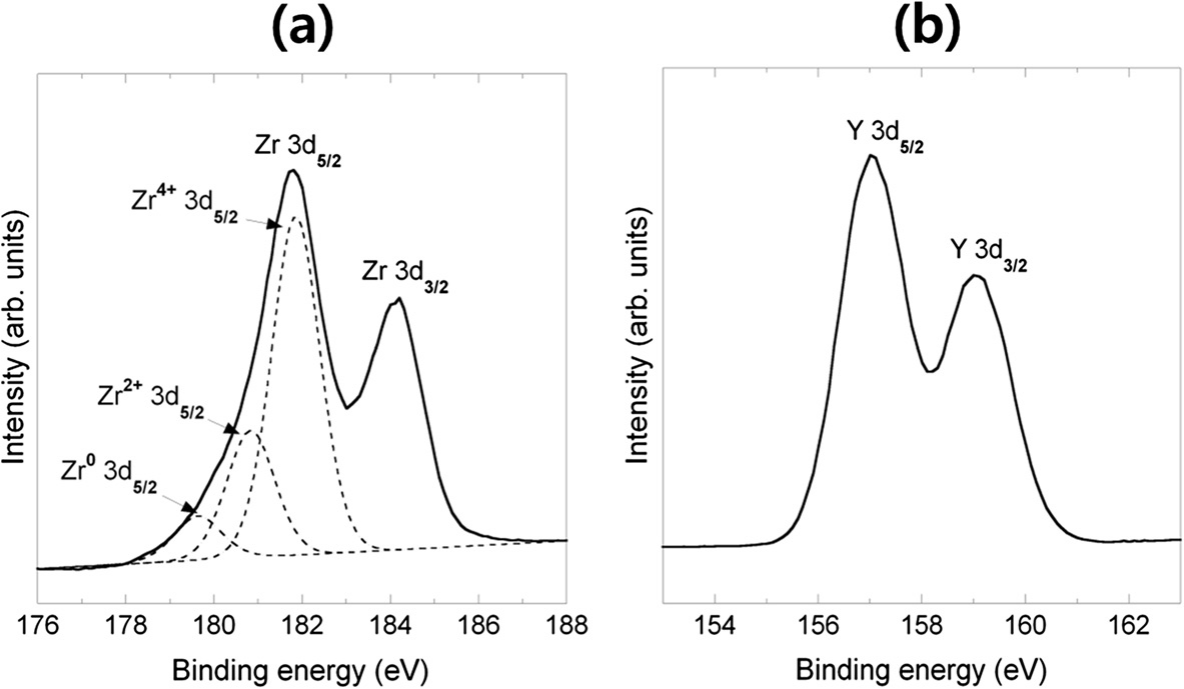
**XPS spectra of (a) Zr 3*d *and (b) Y 3*d *core levels of zirconia/yttria thin films.**

We applied the ALD technique, thus enabling excellent step coverage to fabricate the ultrathin conformal YSZ layer using a commercial ALD system (Plus-100, Quros Co., Ltd., Osan, South Korea) [24,25]. Prior to the deposition of a YSZ thin-film, zirconia and yttria films were separately deposited and characterized for a systematic study. Both films were fabricated by repeating the sequence of precursor pulse (3 s), purge (20 s), oxidant pulse (1 s), and purge (10 s). Tetrakis(dimethylamido)zirconium, Zr(NMe_2_)_4_, and Tris(methylcyclopentadienyl)yttrium, Y(MeCp)_3_, were used as precursors for zirconium and yttrium, respectively. The precursor was delivered using an electropolished stainless steel bubbler fed by Ar gas with 99.99% purity. O_2_ gas was used as the oxidant, and stage temperature was set to 250°C. The temperatures of canisters with charged precursors were 40°C and 180°C, and the line temperatures were 60°C and 210°C for zirconia and yttria deposition, respectively. The growth rates of both zirconia and yttria films during the initial 1,000 cycles were approximately 1 Å/cycle. Although these growth rates were somewhat lower than the reported values (1.2 to 1.5 Å/cycle) [11], the film thickness increased proportionally with the deposition cycles. XPS analyses were performed to determine the chemical composition of an approximately 100-nm-thick zirconia film and an approximately 100-nm-thick yttria film. The atomic concentrations in the zirconia thin-film were as follows: for Zr 3*d*, it was 41.6%, and for O 1*s*, it was 58.4%; they were somewhat different from the expected stoichiometry of ZrO_2_. It is attributed to the fact that reduced zirconium (e.g., Zr^0^ 3d_5/2_ or Zr^2+^ 3d_5/2_) was partially combined with O_2_ during the ALD process, as indicated in the curve fitting result of Figure 2a [26]. The atomic concentrations of the yttria thin-film were Y 3*d* = 40.9% and O 1*s* = 59.1%, which are well aligned with the stoichiometry of Y_2_O_3_. The Y 3*d*_5/2_ peak was located at a binding energy of 156.7 eV, as shown in Figure 2b [27].

Subsequently, YSZ thin films were fabricated by co-deposition of zirconia and yttria. Zirconia was deposited prior to yttria deposition. Yttria mole fraction in the ALD YSZ thin-film was controlled by changing the ratio of deposition cycles for zirconia and yttria. The yttria mole fraction is widely known to determine oxygen ion conductivity in the YSZ, and 8% mole yttria was reported to render the maximum oxygen ion conductivity [1]. When the ratio of zirconia and yttria ALD cycle was 7:1, the atomic concentrations of the YSZ thin-film were as follows: Zr 3*d* = 24.2%, Y 3*d* = 3.6%, and O 1*s* = 72.1%, which were also determined by an XPS analysis. The Y_2_O_3_ mole fraction, *x*, in the YSZ chemical formula of (ZrO_2_)_1−*x*_(Y_2_O_3_)_*x*_ was approximately 0.07. In the case of the YSZ thin-film, the XPS spectra corresponding to an under-stoichiometric ZrO_2_ did not appear, unlike those in the zirconia thin-film.

### Design of AAO-supported GDC/YSZ bilayered thin-film fuel cell

A commercial AAO (Synkera Technology Inc., Longmont, CO, USA) template with an 80-nm pore and a 100-μm height was used as the substrate to leverage their high density of nanopores and resulting electrochemical reaction sites [28,29]. Pt electrode was fabricated by a commercial sputter (A-Tech System Ltd.). Pt with 99.9% purity was used as the Pt target, and the T-S distance was 100 mm. The deposition was conducted at room temperature, and the direct current power was set to 200 W. The Pt anode was deposited on the AAO template in an area of 10 × 10 mm^2^. Dense Pt anodes were deposited at a 5-mTorr Ar pressure, having the growth rate of approximately 60 nm/min. Subsequently, YSZ and GDC electrolytes with an area of 9 × 9 mm^2^ were deposited on the Pt anode. The critical thickness ratio of the YSZ layer to the GDC layer to prevent the reduction of ceria, which was determined considering the distribution of oxygen activity through the thickness of a bilayer, was reported to be approximately 10^−4^ at 800°C and was expected to decrease further at lower temperatures [30]. For this reason, the required minimum thickness of the YSZ layer for electron blockage, if the thickness of GDC layer is 420 nm, is only approximately 0.4 Å. However, a much thicker YSZ film (40 nm) was deposited on the anode side to compensate the rough morphological variations of the Pt-coated AAO surface. The GDC layer, which was 420-nm thick, was then deposited on the YSZ layer. Oxygen reduction reaction happening at the cathode is widely known to cause a significantly greater activation loss compared with the hydrogen oxidation reaction occurring at the anode [1]. In order to facilitate cathode reaction, a porous Pt cathode was prepared by depositing at a much higher Ar pressure of 90 mTorr than that used for anode deposition (5 mTorr Ar). The cathode thickness was approximately 200 nm. The growth rate still remained at approximately 60 nm/min. The Pt cathode, which effectively determines the nominal area of active cell, was deposited using a mask with 1 × 1 mm^2^ openings.

### Electrochemical evaluation of thin-film fuel cells

**Figure 3:**
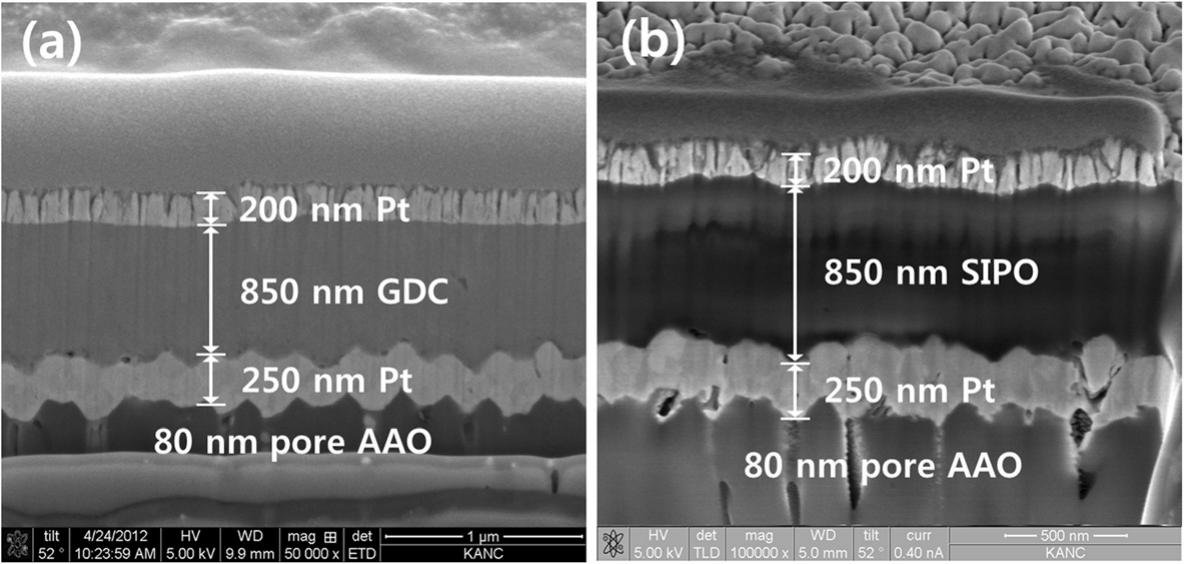
**FE**-**SEM cross**-**sectional images of cells 1 and 2.** (**a**) A GDC single-layered thin-film fuel cell (cell 1) and (**b**) a SIPO single-layered thin-film fuel cell (cell 2).

**Figure 4:**
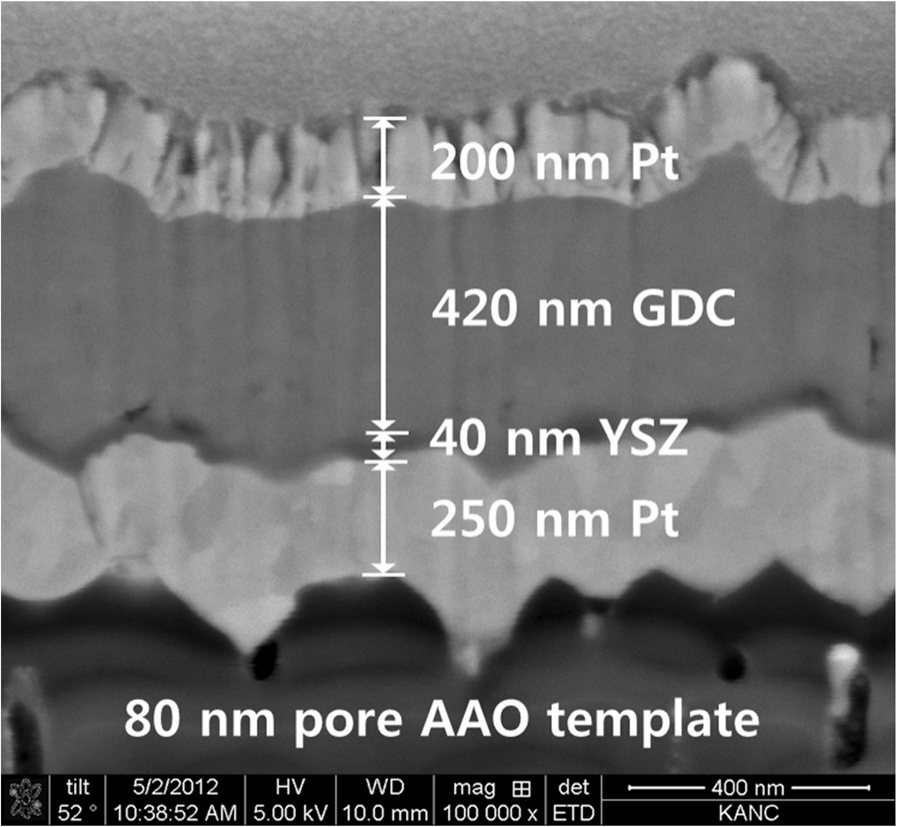
**FE**-**SEM cross**-**sectional image of a GDC**/**YSZ bilayered thin**-**film fuel cell(cell 3).**

**Figure 5:**
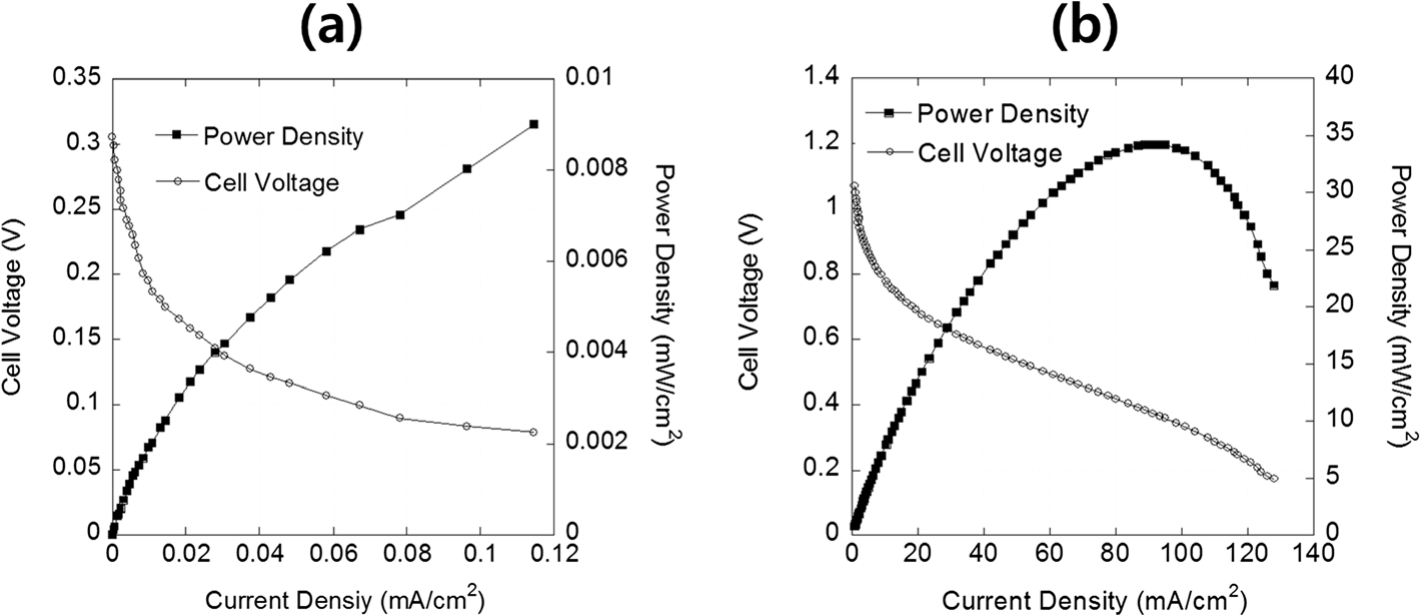
**Electrochemical performances of cells 1 and 3.** (**a**) A 850-nm-thick GDC electrolyte fuel cell (cell 1) and (**b**) a 460-nm-thick GDC/YSZ electrolyte fuel cell (cell 3) measured at 450°C.

Thin-film fuel cells with 850-nm-thick GDC and 850-nm-thick Sn_0.9_In_0.1_P_2_O_7_ (SIPO) electrolytes were fabricated to study further how the ALD YSZ layer have the influence on electrochemical performance [31]. Except for the electrolyte, other cell components were equal to those for GDC/YSZ bilayered thin-film fuel cell. For a comparison with GDC-based cells (cell 1, Pt/GDC/Pt), we fabricated SIPO-based cells (cell 2, Pt/SIPO/Pt). It is postulated that the electrolytes deposited with the same deposition process have identical microstructures [20]. As shown in Figure 3a,b, both the 850-nm-thick dense GDC and SIPO electrolytes did not show any evident pinhole. However, the OCV of approximately 0.3 V for cell 1 was significantly lower than that for cell 2 (approximately 1.0 V). This result indicates that the lower OCV of the GDC-based cells may have originated from oxygen permeation through the GDC electrolyte and/or ceria reduction, not from gas leakage through pinholes. In order to verify the effect of the ALD YSZ layer, we characterized electrochemical performances of GDC/YSZ bilayered thin-film fuel cell (cell 3, Pt/GDC/YSZ/Pt), which has a 40-nm-thick ALD YSZ layer at the anodic interface as shown in Figure 4. As expected, the OCV of cell 3 with the ALD YSZ layer stayed at a decent value of approximately 1.07 V, unlike that of cell 1 (approximately 0.3 V). This discrepancy indicated that the ALD YSZ layer played a successful role as a functional layer to suppress the issues that originated from thin-film GDC electrolyte such as the electronic current leakage and the oxygen permeation [15-17]. The thicknesses of GDC layers in cells 1 and 3 were 850 and 420 nm, respectively. Originally, it was intended for the comparison of the two samples with the same GDC thickness, but a 420-nm-thick GDC-based cell showed highly unstable outputs in the measured quantities. While the peak power density of the cell (cell 3) with an YSZ blocking layer reached approximately 35 mW/cm^2^, that of the single-layered GDC-based cell (cell 1) showed a much lesser power density below approximately 0.01 mW/cm^2^, as shown in Figure 5a,b.

**Figure 6:**
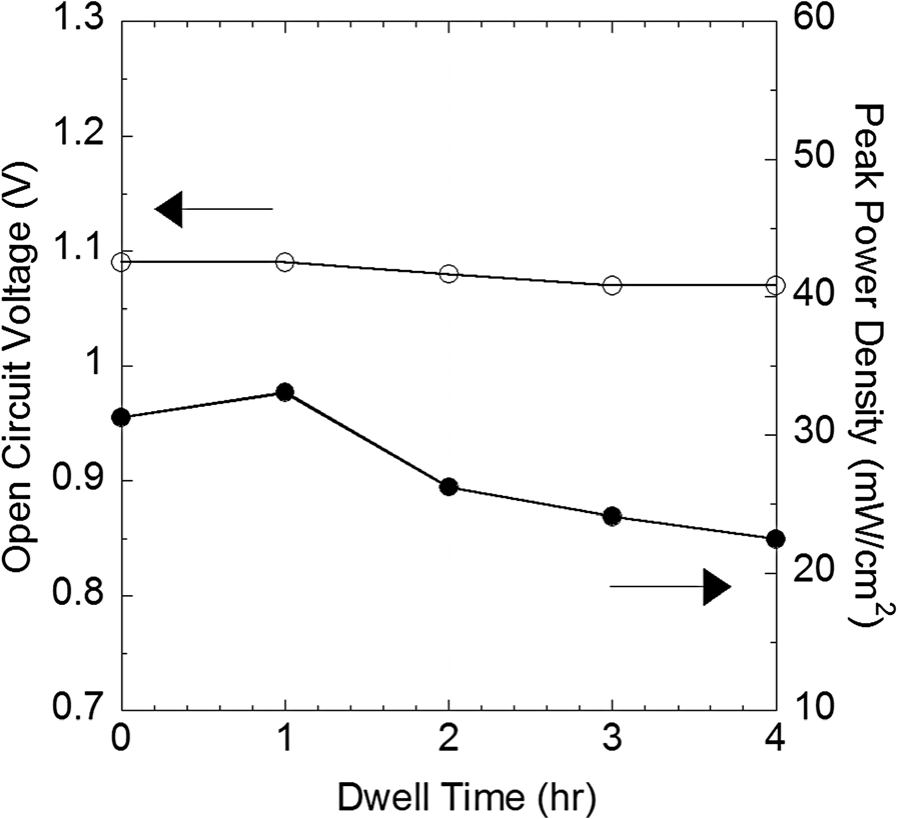
**OCV and peak power density of GDC**/**YSZ thin**-**film fuel cell(cell 3)versus dwell time at 450°C.**

To evaluate the stability of GDC/YSZ bilayered thin-film fuel cell (cell 3), the OCV and the peak power density were measured for 4 h at 450°C, as shown in Figure 6. While reduction of the OCV was negligible, the peak power density sharply decreased by approximately 30% after 4 h. This sharp performance degradation in the AAO-supported thin-film fuel cells was previously studied by Kwon et al. [32]. They ascribed the reason to the agglomeration of the Pt thin-film without microstructural supports. In line with the explanation, the agglomeration of Pt particles was clearly visible when comparing the surface morphologies before and after a cell test, and the degradation of power output caused by the Pt cathode agglomeration was also confirmed through AC impedance measurements. Nevertheless, the stability of AAO-supported GDC/YSZ thin-film fuel cells was relatively superior to ‘freestanding’ thin-film fuel cells with silicon-based substrates [33]. Actually, the configuration of the AAO-supported thin-film fuel cells was maintained after 10 h at 450°C. However, it was reported that freestanding thin-film fuel cells were all broken before 1 h in the same operational conditions [29,33].

## Conclusions

In this study, we implemented and suggested a promising feasibility of a thin-film low-temperature SOFC using a bilayered electrolyte configuration on the AAO platform. GDC has suffered from its chemical instability and the resulting electronic leakage under a reduction environment. In a thin-film configuration for securing a decent oxygen ion conductivity even at low temperatures (as an LT-SOFC), oxygen permeation through the GDC film became problematic as well. This paper reports that an insertion of a very thin ALD YSZ layer between the anode Pt and the GDC electrolyte significantly improved the electrochemical performance of a cell. At 450°C, a thin-film fuel cell with 850-nm-thick GDC electrolyte showed an OCV of approximately 0.3 V and a power density of approximately 0.01 mW/cm^2^. On the other hand, a thin-film fuel cell with a bilayered electrolyte consisting of a 40-nm-thick YSZ and a 420-nm-thick GDC reached an OCV of approximately 1.07 V and a power density of approximately 35 mW/cm^2^. From these results, it was confirmed that the YSZ layer successfully acted as a protective layer. The cell performance is expected to further improve through the microstructural optimization of electrode interfaces and adjustment of chemical compositions of each film.

While the fully functional YSZ layer presented here is already very thin (40 nm), there are good chances of reducing the thickness even further considering that a theoretical approach predicted an YSZ-to-GDC thickness ratio of 0.01% would suffice to guarantee electron blockage [30].

## Competing interests

The authors declare that they have no competing interests.

## Authors’ contributions

SJ designed the experiment, carried out the experimental analysis, and drafted the manuscript. IC and YHL participated in experimental measurements. JP and JYP carried out the growth and optimization of thin-film materials. MHL provided useful suggestions and improve the manuscript. SWC supervised the research work and finalized the manuscript. All authors read and approved the final manuscript.

## Authors’ information

SJ and IC are students in the Graduate School of Convergence Science and Technology, Seoul National University. YHL, JP, and JYP are graduate students in the School of Mechanical and Aerospace Engineering, Seoul National University. MHL is a professor in the School of Engineering at the University of California, Merced. SWC is a professor in the School of Mechanical and Aerospace Engineering, Seoul National University.
